# *Tinggianthura alba*: A New Genus and Species of Anthuridae (Isopoda, Cymothoida, Anthuroidea) from Pulau Tinggi, Johor, Malaysia with an Updated Key to the Genera of Anthuridae

**DOI:** 10.1371/journal.pone.0099072

**Published:** 2014-06-10

**Authors:** Melvin Chew, Azman Abdul Rahim, Othman bin Haji Ross

**Affiliations:** 1 Department of Marine Science, School of Environmental & Natural Resource Sciences, Faculty of Science and Technology, Universiti Kebangsaan Malaysia (UKM), Bangi, Selangor, Malaysia; 2 Marine Ecosystem Research Center (EKOMAR), Faculty of Science and Technology, Universiti Kebangsaan Malaysia (UKM), Bangi, Selangor, Malaysia; University of Waikato (National Institute of Water and Atmospheric Research), New Zealand

## Abstract

A new anthurid isopod from dead coral rubble and stones in the intertidal area of Pulau Tinggi, Johor, Malaysia, is described. It is placed in a new genus and species, *Tinggianthura alba*. *Tinggianthura* is characterized by: (1) subtriangular carpus shape of pereopods 4–7, (2) pereopod 1 propodus palm without prominent tooth or steps and (3) maxillipedal palp 2-articled.

## Introduction

Brandt & Poore [1] reappraised the suborder Anthuridea Leach, 1814 reducing it to superfamily rank, Anthuroidea Leach, 1814 within the suborder, Cymothoida Wägele, 1989. Prior to that revision, a cladistic analysis by Poore [2] resulted in the recognition of six families, four existing (Anthuridae Leach, 1814; Antheluridae Poore & Lewton, 1988; Hyssuridae Wägele, 1981; Paranthuridae Menzies & Glynn, 1968) and two new (Expanathuridae Poore, 2001; Leptanthuridae Poore, 2001). The family Anthuridae, is the oldest and largest family within the Anthuroidea with the most genera recorded. With a new genus *Leipanthura* Poore, 2009 erected lately discovered from the Great Barrier Reef, Australia [3], the number of genera was brought to 25 in total.

According to Myers et al. [4], Malaysia which falls under the Sundaland region has been regarded as one of the hotspot of biodiversity. Despite the fact, Negeoscu [5] reported that the anthuroid isopods from this region are still poorly known. There is no significant attempt to explore the isopod fauna from these waters except the study of Hans-George Müller on the coral reef fauna of two Malaysian islands Pulau Babi Besar and Pulau Tioman over a period of three weeks in April 1991 [6], [7], [8], [9]. In that work, six new species was found, namely *Apanthura bruscai*, *A. tiomanae*, *Eisothistos besar*, *Leptanthura coralliophila*, *Mesanthura asiatica* and *M. kiliani*. Also, four existing species *Apanthura stocki*, *Cyathura bentotae*, *Mesanthura albolineata* and *M. protei* were recorded for the first time from Malaysian waters. In this paper, a new genus is established to accommodate the new species *Tinggianthura alba*.

## Materials and Methods

**Figure 1:**
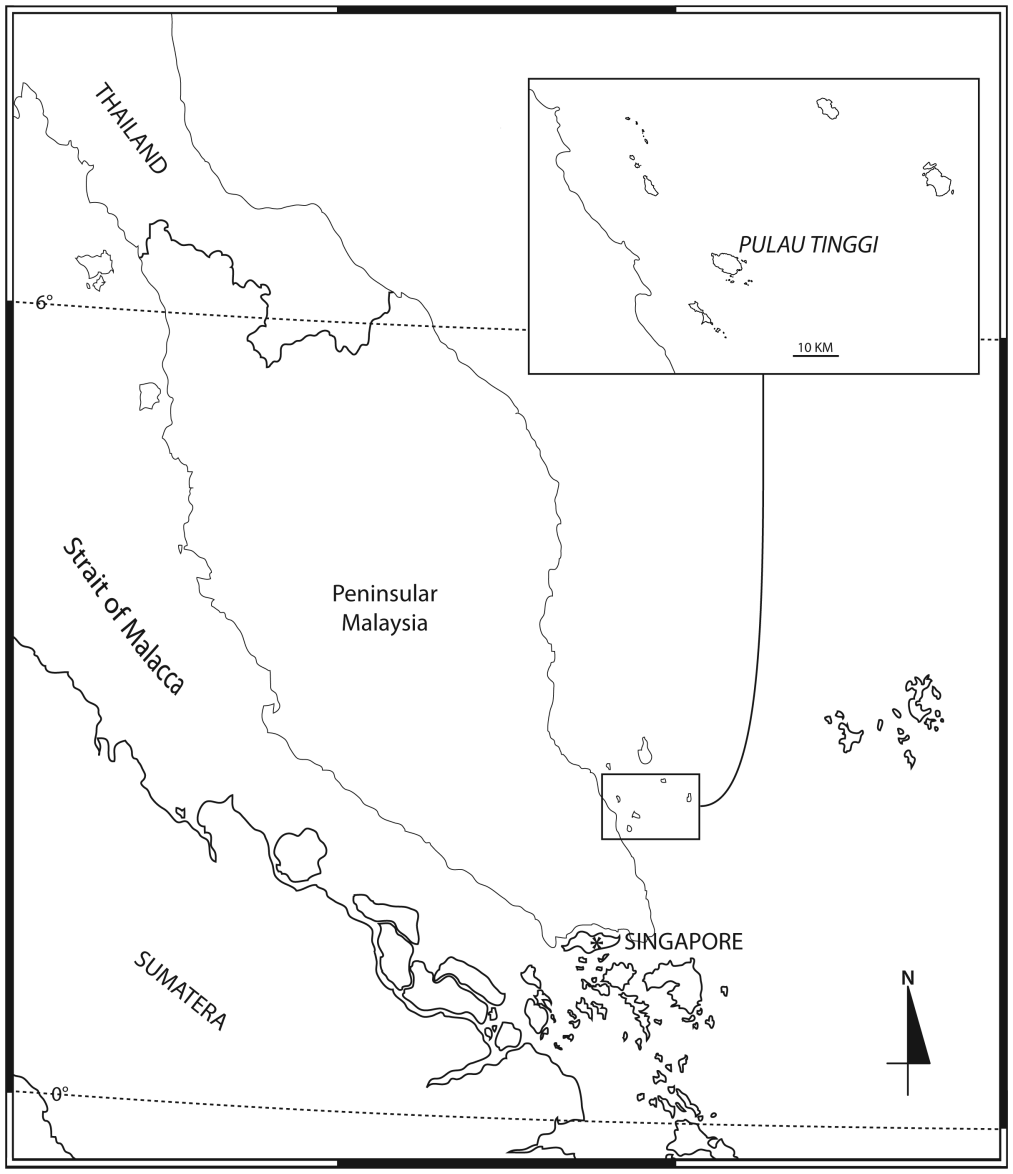
Pulau Tinggi, Johor, Malaysia.

The specimens in this study were obtained from the intertidal area of Pulau Tinggi, Johor located in the southeast coast of Peninsula Malaysia (Figure 1). Samples of dead coral substrate and stones were collected into a bucket with seawater. They are moderately broken up, then a few drops of concentrated formaldehyde were added and left to stand for about 30 minutes. Next, the samples were rinsed with seawater with the washings passed through a 500 µm sieve. In the field, samples were fixed with 10% formalin in sea water. At the laboratory, the specimens were sorted and conserved separately in 4% formalin in water for later examination. Whole body and dissected appendages were mounted in glycerol and illustrated under a Leica DMLB light microscope equipped with a camera lucida.

### Ethics Statement

This study was carried out in strict accordance with the recommendations in Animal Care & Use Guidelines of Universiti Kebangsaan Malaysia Animal Ethics Committee (UKMAEC). The animals studied in this research are microscopic crustaceans found from dead coral rubbles which need no ethics approval from the Universiti Kebangsaan Malaysia Animal Ethics Committee (UKMAEC). Sultan Iskandar Marine Park, Johor has issued the permit for animal collection in Pulau Tinggi, Johor. All efforts were made to minimize suffering and habitat destruction.

### Nomenclatural Acts

The electronic edition of this article conforms to the requirements of the amended International Code of Zoological Nomenclature, and hence the new names contained herein are available under that Code from the electronic edition of this article. This published work and the nomenclatural acts it contains have been registered in ZooBank, the online registration system for the ICZN. The ZooBank LSIDs (Life Science Identifiers) can be resolved and the associated information viewed through any standard web browser by appending the LSID to the prefix "http://zoobank.org/". The LSID for this publication is: urn:lsid:zoobank.org:pub:067317CC-B719-4FF4BCB3-D58335F41452. The electronic edition of this work was published in a journal with an ISSN, and has been archived and is available from the following digital repositories: PubMed Central, LOCKSS.

## Results and Discussion

The new genus could be identified using the following updated key, taken and modified from Poore's (2001) and (2009) key to include *Tinggianthura*.

### Key to the genera of Anthuridae

1. Uropodal exopod terminal, cylindrical.............................*Leipanthura*

- Uropodal exopod subterminal, dorsal, leaf-like..........................2

1. Pereopods 4–7 with carpus twice width of propodus, rectangular, with obvious free distal margin (usually with row of pectinate setae) defined by stout seta on posterodistal angle; maxillipedal endite half length of palp, distally rounded; palp articles 1 and 2 separated by suture (4 or 5 articles visible)........................................................................*Quantathura*

- Pereopods 4–7 with carpus of similar width to propodus, or if wider triangular, only short free distal margin between base of dactylus and stout seta; maxillipedal endite weak or absent, acute if present; palp articles 1–2 and 4–5 fused (maximum of 3 articles visible).............................................................................................3

1. Pereopods 2–7 ischium-propodus bearing long setae on upper and lower margin; blind or eyes weakly pigmented...............................................................................................4

- Pereopods 2–7 ischium-propodus bearing few short setae, most on lower margin (merus-carpus posteriorly lobed and setose in some *Apanthura*); eyes usually pigmented.........................................5

1. Carpus of pereopods 4–7 longer and wider than propodus; mandibular palp of 3 articles.......................................*Notanthura*

- Carpus of pereopods 4–7 shorter than propodus; mandibular palp of 2 articles…...............................................................*Cortezura*

1. Pereopods 4–7 with carpus more or less triangular, upper margin much shorter than lower margin, distal margin oblique and with posterodistal lobe........................................................6

- Pereopods 4–7 with carpus more or less rectangular, upper margin nearly as long as lower margin, distal margin tansverse and without posterodistal lobe.............................................................16

1. Pereopod 4–7 carpus with 1 robust seta on posterodistal angle..........................................................................................7

- Pereopod 4–7 carpus without robust setae on posterior margin or on posterodistal angle...................................................................13

1. Pereopod 1 palm straight without step or prominent tooth.........................................................................*Tinggianthura*

- Pereopod 1 palm with step or prominent tooth................................................................................................8

1. Maxillipedal palp articles 1–2 fused, 3 free and 4–5 fused with terminal articles (4–5) at least one third as long as penultimate article (3)....................................................................................9

- Maxillipedal palp articles 1–2 fused, 3 free and 4–5 fused with terminal article (4–5) minute, without a free mesial margin between its suture and distal group of setae................................10

1. Pereopod 1 chelate, propodus produced posterodistally, dactylus with complexly ridged distal margin, unguis subterminal, carpus and propodus fused.............................*Chelanthura*

- Pereopod 1 subchelate, dactylus and terminal unguis closing on axial palm, carpus and propodus separated by suture...................................................................................*Mesanthura*

1. Maxillipedal palp with fused articles 1–2 longer than broad; mandibles asymmetrical, left molar with tooth fitting socket of right molar; pereopods 2 and 3 with propodus discoid.................................................................................*Apanthuropsis*

- Maxillipedal palp with fused articles 1–2 broader than long; mandibles symmetrical; pereopods 2 and 3 with propodus linear.............................................................................................11

1. Pleonites 1–5 separated by folds dorsally and laterally except dorsally between 4 and 5........................................*Amakusanthura*

- Pleonites 1–5 not separated by folds dorsally..............................12

1. Antenna 2 flagellum with 3–4 articles; male with antenna 1 flagellum of 1 basal +10 aesthetasc-bearing articles, as long as head; head without produced chin except in some males.....................................................................................*Apanthura*

- Antenna 2 flagellum with 1–2 articles; male with antenna 1 flagellum of 1 basal +1 or 2 short aesthetasc-bearing articles, never as long as head; head with produced chin in both sexes.......................................................................................*Skuphonura*

1. Maxillipedal endite absent, palp 1–2 fused, 3 free and 4–5 fused with terminal articles (4–5) at least as long as penultimate article (3)..................................................................................14

- Maxillipedal endite present as triangular lobe, palp articles 1–5 fused..............................................................................................15

1. Uropodal exopod leaf-shaped, articulating along peduncle; marine and estuarine......................................................*Cyathura*

- Uropodal exopod linear, articulating transversely; stygobiontic in Caribbean......................................................................*Stygocyathura*

1. Pleon shorter than wide; mandibular palp of 1 article with long seta.............................................................................*Pendanthura*

- Pleon longer than wide; mandibular palp of 3 articles...................................................................................*Sauranthura*

1. Maxillipedal palp of 3 articles of similar length; maxilliped with endite; mandible with spike-like molar on right side, none of left; pleonite 6 not separated from telson by transverse ridge.........................................................................*Apanthuroides*

- These characters not in combination; mandible with triangular or blunt molar, symmetrical.........................................................17

1. Maxillipedal palp with articles 1–2 fused, 3 free, 4–5 fused or 3–5 fused; endite triangular or absent..........................................................................................18

- Maxillipedal palp with articles 1–3 fused and 4–5 fused or all fused; endite absent......................................................................21

1. Maxillipedal palp with terminal fused articles 4–5 contributing to mesial margin of palp, with setae on mesial margin; telson with longitudinal middorsal ridge; uropodal endopod short, oblique...........................................................................*Idanthura*

- Maxillipedal palp with terminal fused articles 4–5 minute, triangular or semicircular, not contributing to mesial margin of palp, with setae on distal or distomesial margin; telson broadly rounded, rarely with longitudinal middorsal ridge; uropodal endopod at least as long as wide, its suture with peduncle more or less transverse..........................................................................19

1. Pereopod 1 merus barely touching cylindrical propodus on anterior margin; maxillipedal endite absent; male antenna 1 of 10 articules...................................................................*Cetanthura*

- Pereopod 1 merus cupping swollen subchelate propodus; maxillipedal endite present; male antenna 1 of 20 articles................................................................................................20

1. Body covered at least in part with setules; pleonites 1–5 with sutures indicated dorsally; antenna 2 flagellum of 7 articles; eyes absent.................................................................*Pilosanthura*

- Body smooth, without setules; pleonites 1–5 without sutures indicated dorsally; antenna 2 flagellum of 5 articles; eyes present...........................................................................*Malacanthura*

1. Pereopod 1 palm with strong proximal seta (about as long as unguis); maxillipedal palp with fused articles 4–5 small, transverse, lateral to distomesial lobe of fused articles 1–3; mandibular palp of 1 or 2 artilces.............................*Caenanthura*

- Pereopod 1 palm without strong proximal seta; maxillipedal palp with fused articles 4–5 oblique; mandibular palp of 3 articles................................................................................................22

1. Maxillipedal palp with articles 1–3 fused and 4–5 fused.........................................................................................23

- Maxillipedal palp with articles 1–5 fused.................................24

1. Pereopod 1 propodus swollen, subchelate; dactylus simple and closing on palm; body of usually propotions, rarely slender.............................................................................*Haliophasma*

- Pereopod 1 propodus cylindrical; dactylus with teeth along lower margin, not closing on palm; body very slender....................................................................................*Nemanthura*

1. Mandibular palp of 3 articles.........................................*Anthura*

- Mandibular palp of 1 article......................................................25

1. Pereopod 7 present.....................................................*Ptilanthura*

- Pereopod 7 absent........................................................*Exallanthura*

### Systematics

**Order ISOPODA Latreille, 1817**

**Suborder CYMOTHOIDA Wägele, 1989**

**Family Anthuridae Leach, 1814**

***Tinggianthura gen. n.*** *urn:lsid:zoobank.org:act:FF6B58D5-0515-4BC7-B64D-2F3222350DCA*

#### Diagnosis

Body not pigmented, smooth. Pleonites 1–5 together as long as greatest width, fused. Antenna 2 flagellum of 2 articles, shorter than article 5 of peduncle. Mandibular palp of 3 articles with article 3 shorter than article 1. Maxillipedal endite absent; palp articles 1–3 fused, 4–5 fused; palp article 3 barely produced medially beyond articles 4–5; palp terminal articles (4–5) oblique, without a free mesial margin between its suture and distal group of setae. Pereopod 1 propodus palm straight, without prominent tooth or strong proximal seta. Pereopods 4–7 carpus more or less triangular with 1 robust seta on posterodistal angle.

*Male*: Antenna 1 flagellum of 4 articles with 1 aesthetasc-bearing article.

#### Type species

*Tinggianthura alba*, new species, here designated.

#### Etymology

Named after the type locality Pulau Tinggi in combination with the ‘*anthura*’ stem.

#### Remarks

The new genus shares maxillipedal palp with a single suture with *Caenanthura* Kensley, 1978, *Haliophasma* Haswell, 1881, *Nemanthura* Wägele, 1981 and *Notanthura* Monod, 1927.

The new genus is similar to *Caenanthura* Kensley, 1978 in having short flagella of antenna 2, pereopods 4–7 carpus shape, straight propodal palm of pereopod 1 and the 2-articled maxillipedal palp. However, the pereopod 1 propodus palm in *Caenanthura* exhibit a strong proximal seta. Apart from this, *Tinggianthura* differs in having mandibular palp of 3 articles compared to 1 or 2 very short articles in *Caenanthura*.

The pereopods 4–7 carpus more or less triangular in *Tinggianthura* separates this genus from *Haliophasma*, *Nemanthura* and *Notanthura* having their pereopods 4–7 carpus more or less rectangular.

**Figure 2:**
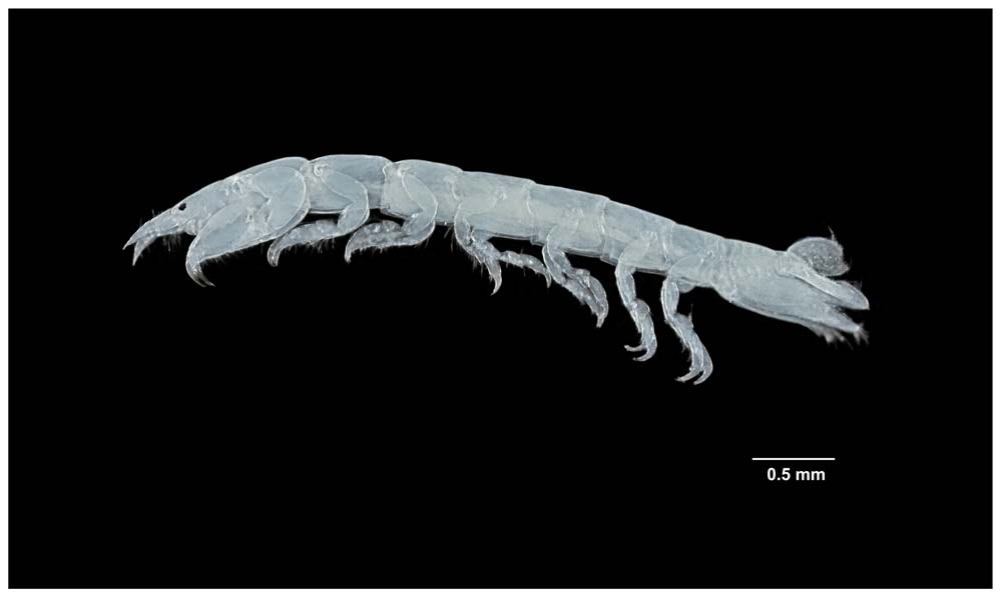
*Tinggianthura alba* sp. n., holotype, female, (UKMMZ-1479).

***Tinggianthura alba sp. n.*** *urn:lsid:zoobank.org:act:042EB2FA-D803-4840-92AF-A33C51446474 (* *Figure 2B* *)*

#### Type Material

Holotype. Female, UKMMZ-1479 (Figure 2) Malaysia, Johor, Pulau Tinggi, Kampung Pasir Panjang, 2°17'35.08"N, 104° 6'7.13"E, C.W.H. Melvin, 16 August 2012, coral rubbles.

Allotype. Male, UKMMZ-1480

Paratypes. 2 males, 12 females, 1 juvenile, UKMMZ-1481; 2 males, 12 females, 1 juvenile, UKMMZ-1482; 2 males, 12 females, 1 juvenile,UKMMZ-1483.

#### Etymology

*alba*, from the Latin meaning white, in allusion to the whitish body colour of the specimens.

#### Description

holotype adult female.

**Figure 3:**
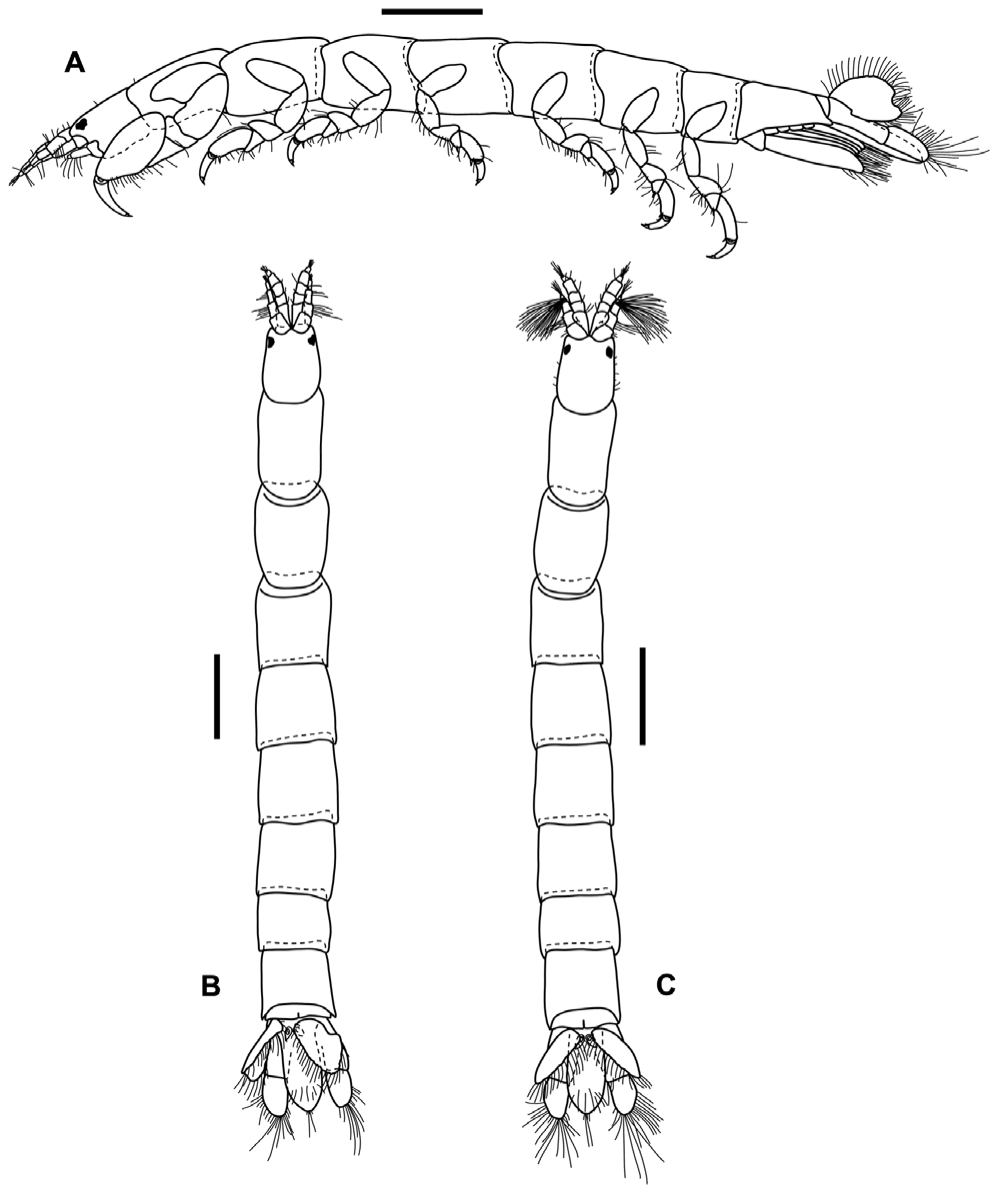
*Tinggianthura alba* sp. n. All scales represent 0.5(A) holotype female lateral (UKMMZ-1479), (B) holotype female dorsal (UKMMZ-1479), (C) allotype male dorsal (UKMMZ-1480).

Total body length 3.9 mm (tip of rostrum to base of pleotelson), approximately 8.7 times longer than greatest width; pale white colour, pigmentless. Head longer than wide, smooth, with a minute rostrum; eyes present, small. Pereonites smooth, of equal width, pereonites 1–2 longer than wide with pereonite 1 longest, pereonites 3–6 about as long as wide, pereonite 7 shorter than width. Pleonites 1–5 fused, smooth, pleonite 6 dorsally with medial incision on distal margin (Figures 3A, 3B).

**Figure 4:**
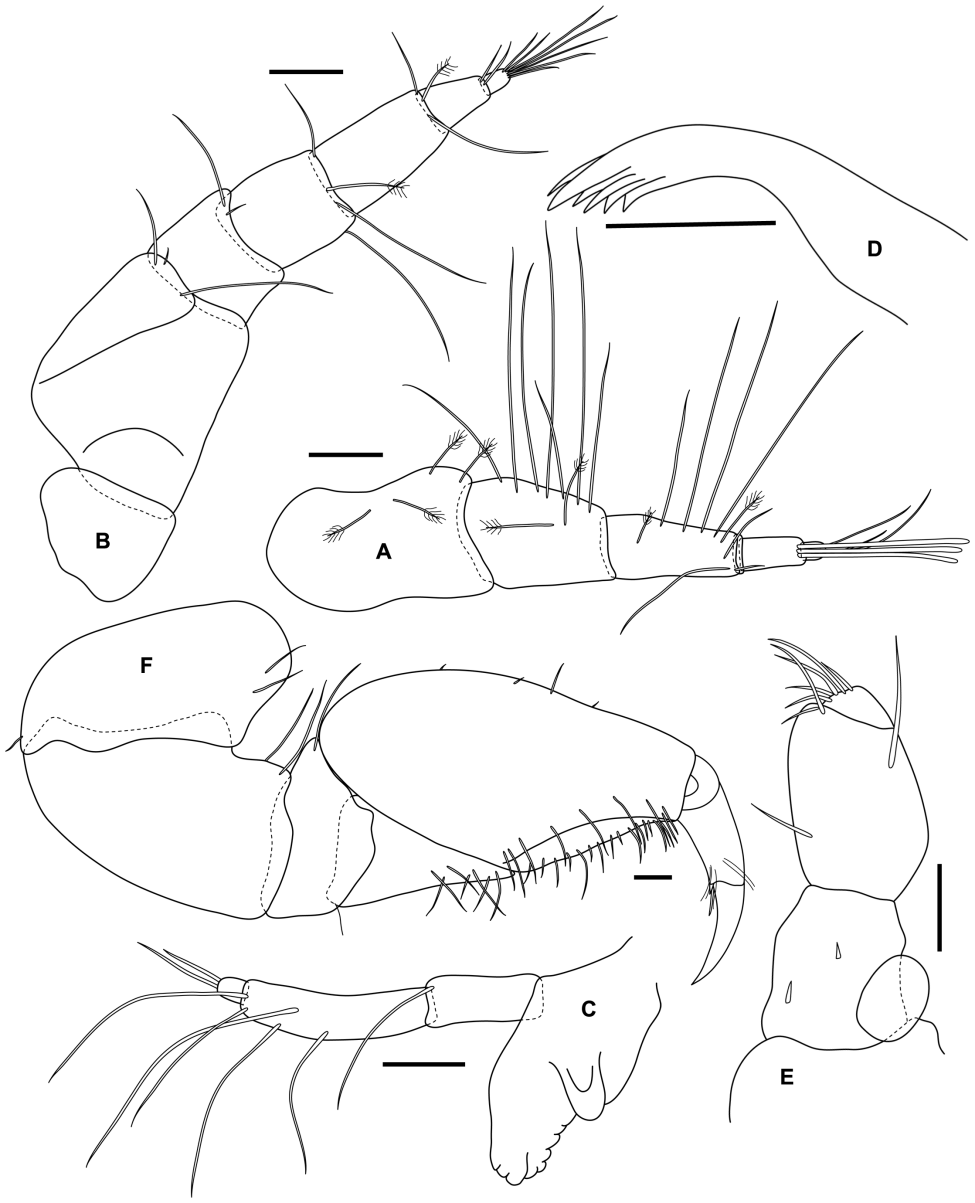
*Tinggianthura alba* sp. n., holotype, female, (UKMMZ-1479). All scales represent 0.05(A) antenna 1, (B) antenna 2, (C) mandible, (D) maxilla, (E) maxilliped, (F) pereopod 1.

Antenna 1 peduncle longer than flagellum, article 1 stout, progressively narrower articles 2 and 3; flagellum of 3 articles shorter that peduncle article 3, article 1 very short almost hidden, article 2 longest with 3 aesthetascs distally, article 3 minute with 3 setae terminally (Figure 4A).

Antenna 2 peduncle longer than peduncle of antenna 1, article 1 subtriangular, article 2 longest and grooved to accommodate Antenna 1, article 5 slightly shorter than article 3 and 4 combined; flagellum of 2 articles with article 1 being the longest, article 2 bearing 7 setae apically (Figure 4B).

Mandibular incisor with 3 teeth; lamina dentate about 6 serrations; molar process rounded tubercle; palp of 3 articles, article 1 with 1 seta distally, article 2 longest with 2 distal setae, 2 mesiodistal setae and 1 mesial seta, article 3 shortest with 2 setae apically (Figure 4C).

Maxilla with strong distal spine and 5 more slender subterminal spines (Figure 4D).

Maxillipedal palp fused article 1–3 with 1 proximal seta, 1 mesial seta and 2 distal setae, fused article 4–5 obliquely set distolaterally on fused article 1–3, about 3.5 times as long as penultimate article, terminally 5 setae (Figure 4E).

Pereopod 1 robust, subchelate; carpus triangular with 7 setae on lower margin; propodus inflated, palm slightly convex with continuous rows of setae; unguis about half length of dactylus (Figure 4F).

**Figure 5:**
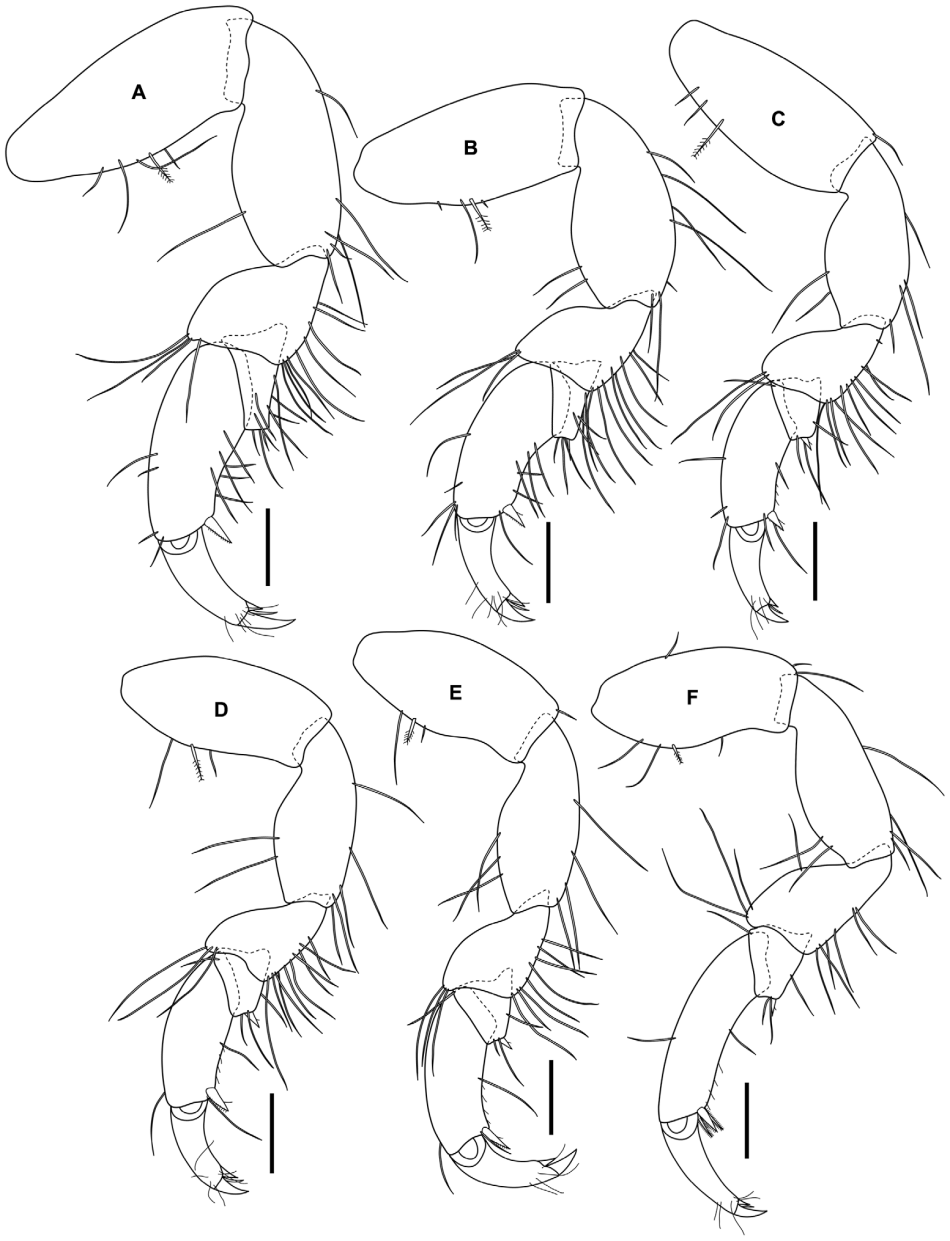
*Tinggianthura alba* sp. n., holotype, female, (UKMMZ-1479). All scales represent 0.1(A) pereopod 2, (B) pereopod 3, (C) pereopod 4, (D) pereopod 5, (E) pereopod 6, (F) pereopod 7.

Pereopod 2 basis about 2.5 times longer than wide; merus with convex upper margin, highly setose on lower margin; carpus subtriangular, highly setose on lower margin; propodus palm slightly concave with 6 setae and 1 distal serrulated robust seta; unguis about one third of dactylus (Figure 5A).

Pereopod 3 similar to pereopod 2, slightly smaller (Figure 5B).

Pereopod 4–6 similar in form to each other, about as long as pereopod 2, basis with 1 plumose seta medially on upper margin; merus with convex upper margin, highly setose on lower margin; carpus subtriangular with 1 robust seta on posterodistal angle; propodus curved with 1 posterodistal serrulated strong seta, palm concave with palmar comb setae and 1 seta each on medial and distal margin; dactylus curved, unguis about one third of dactylus (Figures 5C-5E).

Pereopod 7 similar to pereopod 4–6 except propodus slightly longer and palm with 2 leaf-like denticulate setae posterodistally (Figure 5F).

**Figure 6:**
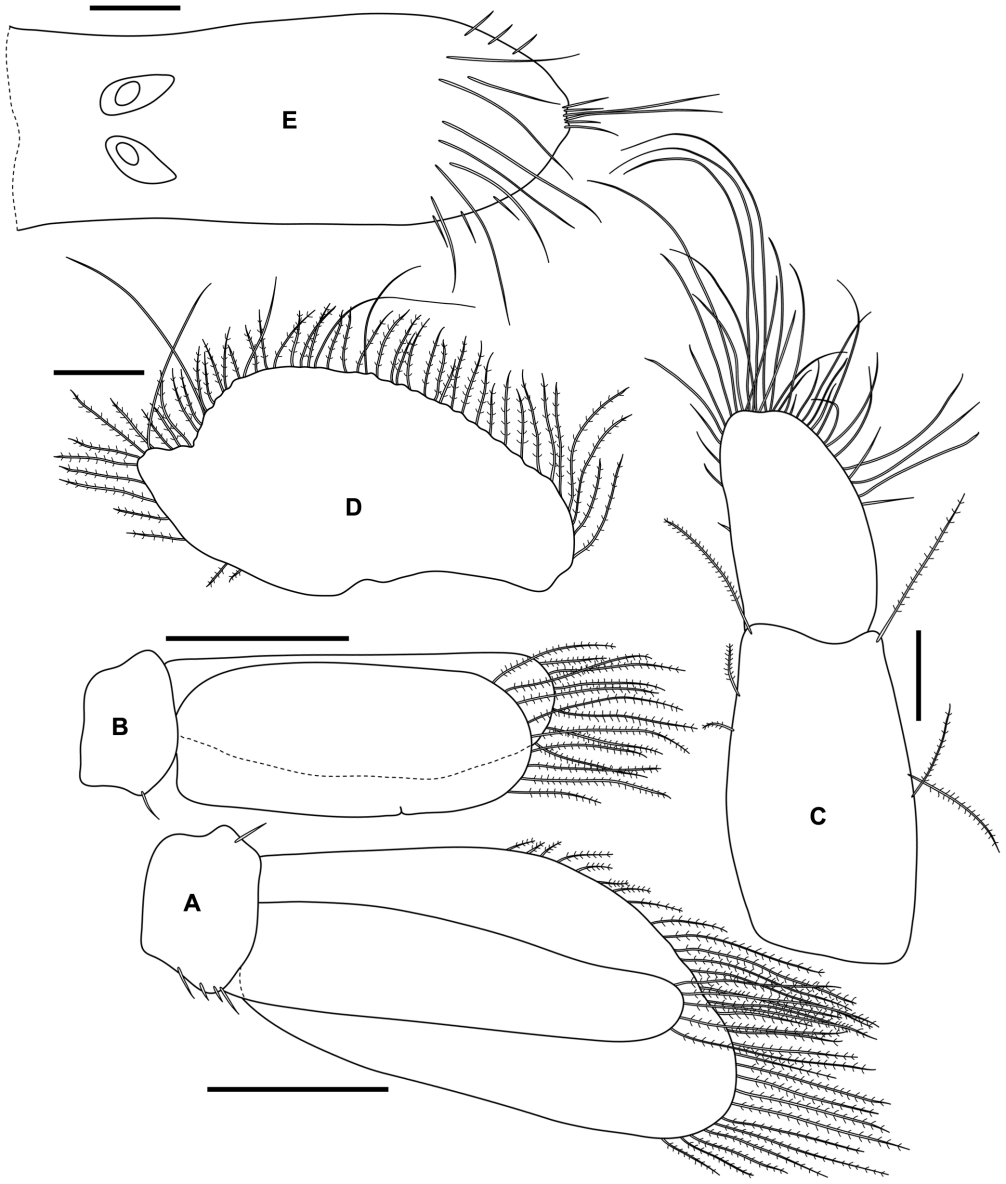
*Tinggianthura alba* sp. n., holotype, female, (UKMMZ-1479). All scales represent 0.1(A) pleopod 1, (B) pleopod 2, (C) uropodal endopod, (D) uropodal exopod, (E) pleotelson.

Pleopod 1 sympod subquadrate; exopod operculiform, about 2 times longer than greatest width, 29 plumose setae on distal margin; endopod narrow, about 4 times longer than greatest width not reaching apex of exopod, 5 plumose setae on distal margin (Figure 6A).

Pleopod 2 sympod subquadrate; exopod about 2.3 times longer than greatest width with 9 plumose setae on distal margin, slightly cleft medially; endopod narrower than exopod about 3.1 times longer than greatest width, distally surrounded by 6 plumose setae (Figure 6B).

Uropod sympod elongate, rectangular; endopod margin ovate with numerous setae (Figure 6C); exopod ovate with strong distal emargination, about twice as long as wide, margin bearing several long setae and numerous plumose setae (Figure 6D).

Pleotelson longer than wide with a pair of statocyst proximally, widest subdistally then tapering to a slightly concave apex bearing a pair of long setae and 2 pairs of short setae, 3 short setae on each border subdistally, subdistal region with 4 pairs of long setae dorsally (Figure 6E).

#### Description

Allotype adult male.

Total body length 3.6 mm, about 9.3 times longer than greatest width; pale white colour, pigmentless. Head, pereon, pleon and pleotelson similar to female (Figure 3C).

**Figure 7:**
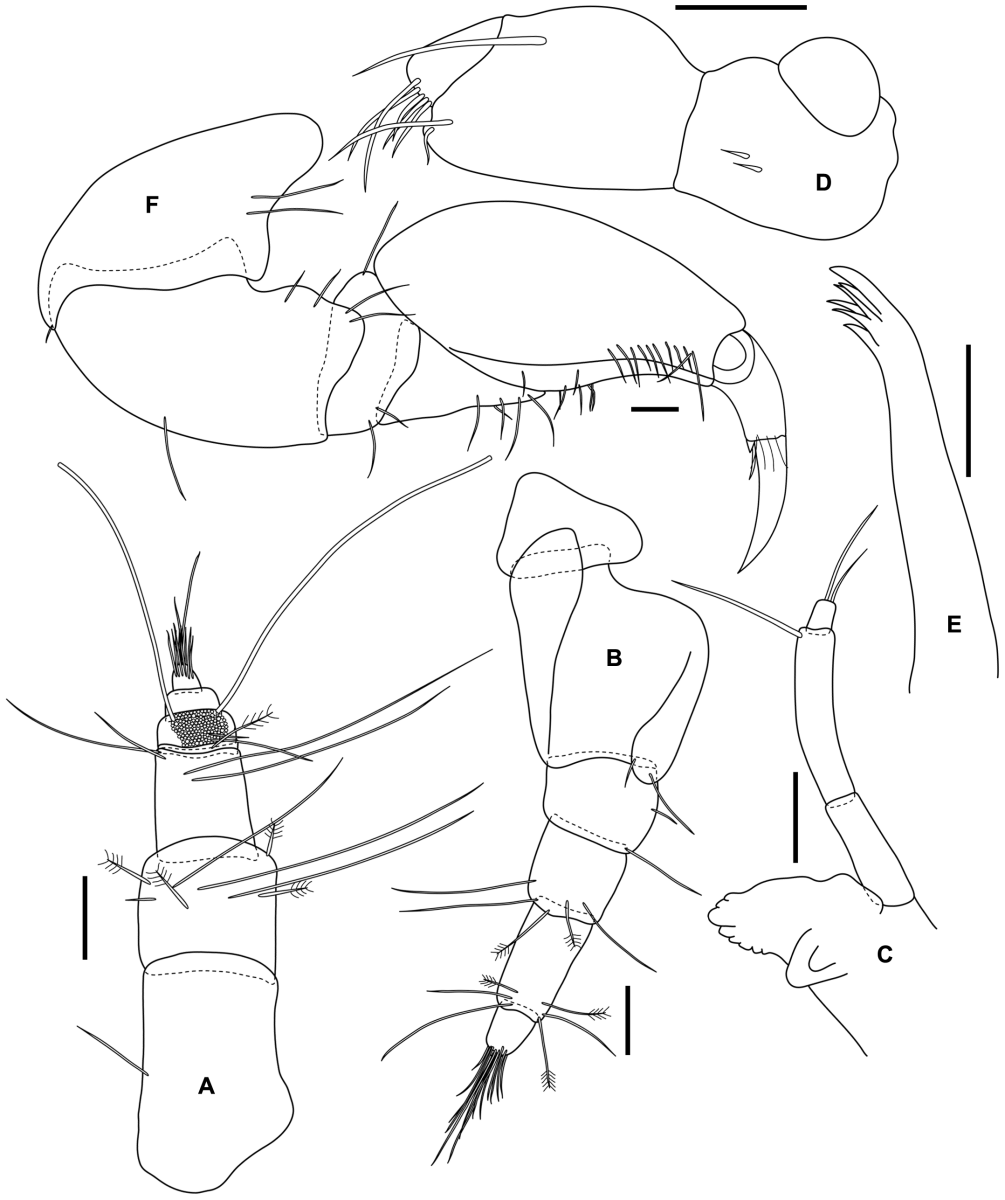
*Tinggianthura alba* sp. n., allotype, male, (UKMMZ-1480). All scales represent 0.05(A) antenna 1, (B) antenna 2, (C) mandible, (D) maxilla, (E) maxilliped, (F) pereopod 1.

Antenna 1 peduncle article 1 robust longer than wide, article 2 slightly wider than long, article 3 narrower; flagellum of 4 articles with article 1 shortest almost hidden, article 2 bearing numerous long aesthetascs particularly on one side, terminal article with about 8 setae apically (Figure 7A).

Antenna 2 similar to female, flagellum article 2 minute hidden by many setae emerging distally from article 1 (Figure 7B).

Mandible similar to female, but with lamina dentate 5 serrations; molar; palp less setose compare to female, article 2 longest with 1 seta distally, article 3 shortest with 2 setae apically (Figure 7C).

**Figure 8:**
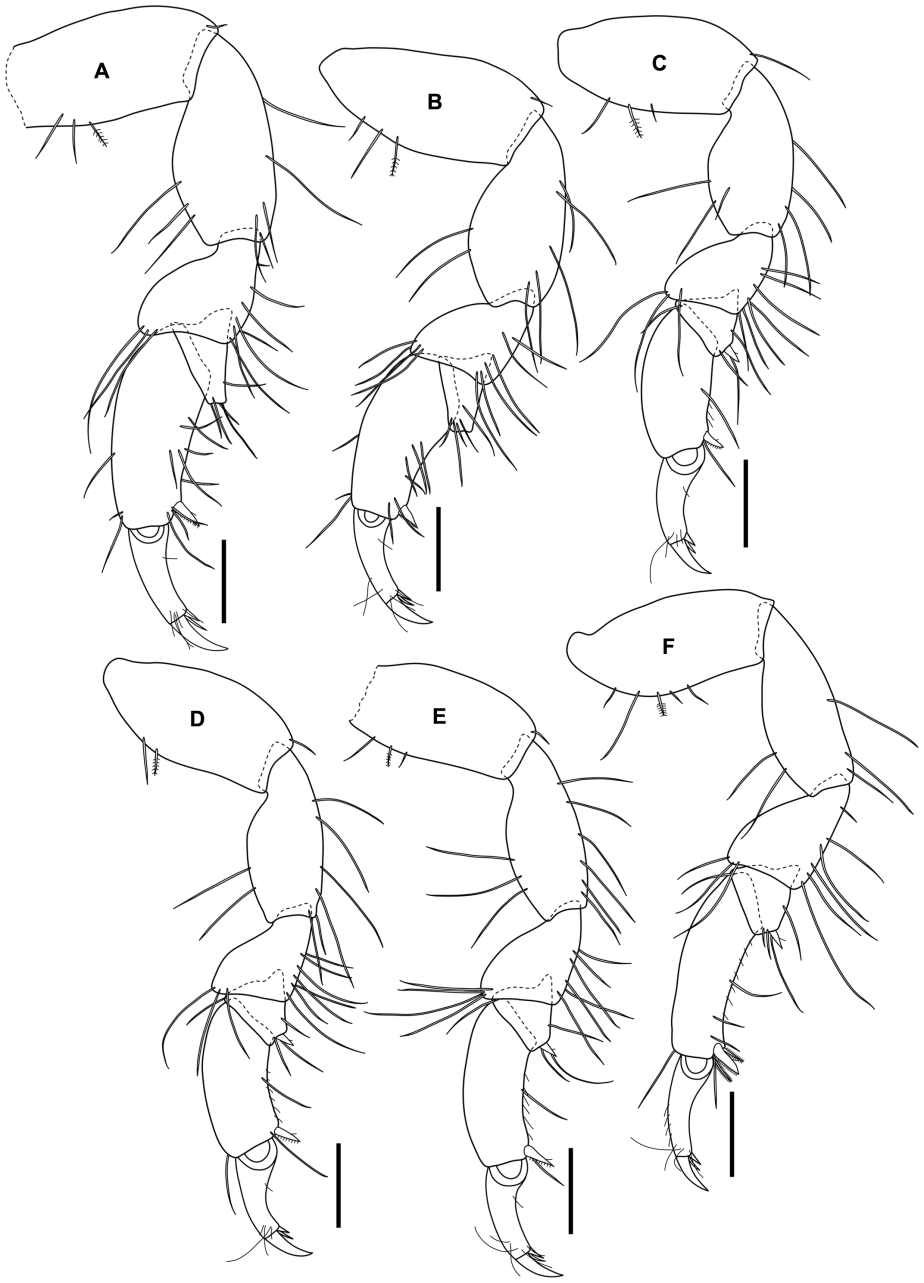
*Tinggianthura alba* sp. n., allotype, male, (UKMMZ-1480). All scales represent 0.1(A) pereopod 2, (B) pereopod 3, (C) pereopod 4, (D) pereopod 5, (E) pereopod 6, (F) pereopod 7.

Maxilla, maxilliped and pereopods 1–7 similar to female (Figures 7D–7F, 8A–8F).

**Figure 9:**
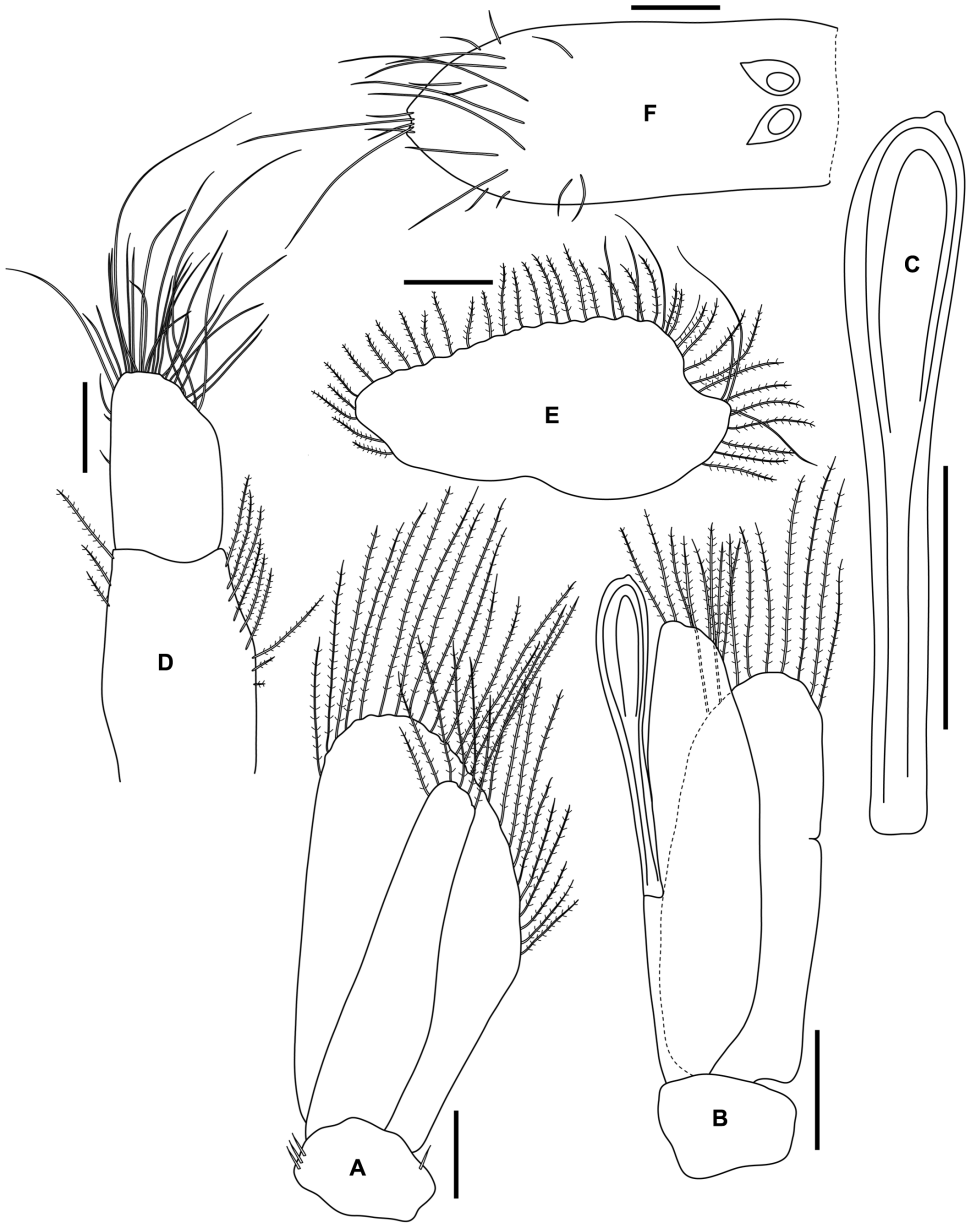
*Tinggianthura alba* sp. n., allotype, male, (UKMMZ-1480). All scales represent 0.1(A) pleopod 1, (B) pleopod 2, (C) appendix masculina, (D) uropodal endopod, (E) uropodal exopod, (F) pleotelson.

Pleopod 1 exopod with 25 plumose setae on distal margin and medially slightly cleft; endopod with 6 plumose setae on distal margin (Figure 9A).

Pleopod 2 exopod with 9 plumose setae on distal margin; endopod with 6 plumose setae on distal margin (Figure 9B); appendix masculina spatulate with a minute protuberance on distal margin (Figures 9C).

Uropod and pleotelson similar to female (Figures 9D–9F).
