# Correction: *Tinggianthura alba*: A New Genus and Species of Anthuridae (Isopoda, Cymothoida, Anthuroidea) from Pulau Tinggi, Johor, Malaysia with an Updated Key to the Genera of Anthuridae

**DOI:** 10.1371/journal.pone.0107419

**Published:** 2014-09-02

**Authors:** The PLOS ONE Staff

The Key to the genera of Anthuridae section in the Results and Discussion is numbered incorrectly. Please see the correct Key to the genera of Anthuridae below.

1. Uropodal exopod terminal, cylindrical.................................................................*Leipanthura*

- Uropodal exopod subterminal, dorsal, leaf-like.......................................................................2

2. Pereopods 4-7 with carpus twice width of propodus, rectangular, with obvious free distal margin (usually with row of pectinate setae) defined by stout seta on posterodistal angle; maxillipedal endite half length of palp, distally rounded; palp articles 1 and 2 separated by suture (4 or 5 articles visible)............................................................................*Quantathura*

- Pereopods 4-7 with carpus of similar width to propodus, or if wider triangular, only short free distal margin between base of dactylus and stout seta; maxillipedal endite weak or absent, acute if present; palp articles 1-2 and 4-5 fused (maximum of 3 articles visible).........3

3. Pereopods 2-7 ischium-propodus bearing long setae on upper and lower margin; blind or eyes weakly pigmented.........................................................................................4

- Pereopods 2-7 ischium-propodus bearing few short setae, most on lower margin (merus-carpus posteriorly lobed and setose in some *Apanthura*); eyes usually pigmented...................5

4. Carpus of pereopods 4-7 longer and wider than propodus; mandibular palp of 3 articles ..........................................*Notanthura*

- Carpus of pereopods 4-7 shorter than propodus; mandibular palp of 2 articles........*Cortezura*

5. Pereopods 4-7 with carpus more or less triangular, upper margin much shorter than lower margin, distal margin oblique and with posterodistal lobe........................................................6

- Pereopods 4-7 with carpus more or less rectangular, upper margin nearly as long as lower margin, distal margin tansverse and without posterodistal lobe..............................................16

6. Pereopod 4-7 carpus with 1 robust seta on posterodistal angle..............................................7

- Pereopod 4-7 carpus without robust setae on posterior margin or on posterodistal angle.....13

7. Pereopod 1 palm straight without step or prominent tooth...............................*Tinggianthura*

- Pereopod 1 palm with step or prominent tooth........................................................................8

8. Maxillipedal palp articles 1-2 fused, 3 free and 4-5 fused with terminal articles (4-5) at least one third as long as penultimate article (3)........................................................................9

- Maxillipedal palp articles 1-2 fused, 3 free and 4-5 fused with terminal article (4-5) minute, without a free mesial margin between its suture and distal group of setae..............................10

9. Pereopod 1 chelate, propodus produced posterodistally, dactylus with complexly ridged distal margin, unguis subterminal, carpus and propodus fused...............................*Chelanthura*

- Pereopod 1 subchelate, dactylus and terminal unguis closing on axial palm, carpus and propodus separated by suture...............................................................................*Mesanthura*

10. Maxillipedal palp with fused articles 1-2 longer than broad; mandibles asymmetrical, left molar with tooth fitting socket of right molar; pereopods 2 and 3 with propodus discoid..........................................................................*Apanthuropsis*

- Maxillipedal palp with fused articles 1-2 broader than long; mandibles symmetrical; pereopods 2 and 3 with propodus linear...................................................................................11

11. Pleonites 1-5 separated by folds dorsally and laterally except dorsally between 4 and 5................................*Amakusanthura*

- Pleonites 1-5 not separated by folds dorsally.........................................................................12

12. Antenna 2 flagellum with 3-4 articles; male with antenna 1 flagellum of 1 basal + 10 aesthetasc-bearing articles, as long as head; head without produced chin except in some males.............................................................................*Apanthura*

- Antenna 2 flagellum with 1-2 articles; male with antenna 1 flagellum of 1 basal + 1 or 2 short aesthetasc-bearing articles, never as long as head; head with produced chin in both sexes...................................................................*Skuphonura*

13. Maxillipedal endite absent, palp 1-2 fused, 3 free and 4-5 fused with terminal articles (4-5) at least as long as penultimate article (3).............................................................................14

- Maxillipedal endite present as triangular lobe, palp articles 1-5 fused..................................15

14. Uropodal exopod leaf-shaped, articulating along peduncle; marine and estuarine.........................................................*Cyathura*

- Uropodal exopod linear, articulating transversely; stygobiontic in Caribbean.................................................*Stygocyathura*

15. Pleon shorter than wide; mandibular palp of 1 article with long seta...............*Pendanthura*

- Pleon longer than wide; mandibular palp of 3 articles..........................................*Sauranthura*

16. Maxillipedal palp of 3 articles of similar length; maxilliped with endite; mandible with spike-like molar on right side, none of left; pleonite 6 not separated from telson by transverse ridge.................................................*Apanthuroides*

- These characters not in combination; mandible with triangular or blunt molar, symmetrical.........................................................17

17. Maxillipedal palp with articles 1-2 fused, 3 free, 4-5 fused or 3-5 fused; endite triangular or absent.................................18

- Maxillipedal palp with articles 1-3 fused and 4-5 fused or all fused; endite absent..............21

18. Maxillipedal palp with terminal fused articles 4-5 contributing to mesial margin of palp, with setae on mesial margin; telson with longitudinal middorsal ridge; uropodal endopod short, oblique...........................................................................*Idanthura*

- Maxillipedal palp with terminal fused articles 4-5 minute, triangular or semicircular, not contributing to mesial margin of palp, with setae on distal or distomesial margin; telson broadly rounded, rarely with longitudinal middorsal ridge; uropodal endopod at least as long as wide, its suture with peduncle more or less transverse........................................................19

19. Pereopod 1 merus barely touching cylindrical propodus on anterior margin; maxillipedal endite absent; male antenna 1 of 10 articules.............................................................*Cetanthura*

- Pereopod 1 merus cupping swollen subchelate propodus; maxillipedal endite present; male antenna 1 of 20 articles..............................................................20

20. Body covered at least in part with setules; pleonites 1-5 with sutures indicated dorsally; antenna 2 flagellum of 7 articles; eyes absent.........................................................*Pilosanthura*

- Body smooth, without setules; pleonites 1-5 without sutures indicated dorsally; antenna 2 flagellum of 5 articles; eyes present......................................................................*Malacanthura*

21. Pereopod 1 palm with strong proximal seta (about as long as unguis); maxillipedal palp with fused articles 4-5 small, transverse, lateral to distomesial lobe of fused articles 1-3; mandibular palp of 1 or 2 artilces............................................................................*Caenanthura*

- Pereopod 1 palm without strong proximal seta; maxillipedal palp with fused articles 4-5 oblique; mandibular palp of 3 articles......................................................................................22

22. Maxillipedal palp with articles 1-3 fused and 4-5 fused....................................................23

- Maxillipedal palp with articles 1-5 fused...............................................................................24

23. Pereopod 1 propodus swollen, subchelate; dactylus simple and closing on palm; body of usually propotions, rarely slender..................................................................*Haliophasma*

- Pereopod 1 propodus cylindrical; dactylus with teeth along lower margin, not closing on palm; body very slender............................................................................*Nemanthura*

24. Mandibular palp of 3 articles...............................................................................*Anthura*

- Mandibular palp of 1 article..........................................................................................25

25. Pereopod 7 present.......................................................................................................................*Ptilanthura*

- Pereopod 7 absent.....................................................................................................................................*Exallanthura*
